# Carbon Dot Micelles Synthesized from Leek Seeds in Applications for Cobalt (II) Sensing, Metal Ion Removal, and Cancer Therapy

**DOI:** 10.3390/jfb15110347

**Published:** 2024-11-15

**Authors:** Teh-Hua Tsai, Wei Lo, Hsiu-Yun Wang, Tsung-Lin Tsai

**Affiliations:** 1 Department of Chemical Engineering and Biotechnology, National Taipei University of Technology, Taipei 106344, Taiwan; thtsai@ntut.edu.tw; 2 Department of Oncology, National Cheng Kung University Hospital, College of Medicine, National Cheng Kung University, Tainan 701401, Taiwan; n321728@gmail.com; 3 Center of Applied Nanomedicine, National Cheng Kung University, Tainan 701401, Taiwan

**Keywords:** carbon dots, supercritical fluid extraction, leek seeds, micelles, cobalt (II) sensor, metal ion removal, cancer therapy

## Abstract

Popular photoluminescent (PL) nanomaterials, such as carbon dots, have attracted substantial attention from scientists due to their photophysical properties, biocompatibility, low cost, and diverse applicability. Carbon dots have been used in sensors, cell imaging, and cancer therapy. Leek seeds with anticancer, antimicrobial, and antioxidant functions serve as traditional Chinese medicine. However, leek seeds have not been studied as a precursor of carbon dots. In this study, leek seeds underwent a supercritical fluid extraction process. Leek seed extract was obtained and then carbonized using a dry heating method, followed by hydrolysis to form carbon dot micelles (CD-micelles). CD-micelles exhibited analyte-induced PL quenching against Co^2+^ through the static quenching mechanism, with the formation of self-assembled Co^2+^-CD-micelle sphere particles. In addition, CD-micelles extracted metal ion through liquid–liquid extraction, with removal efficiencies of >90% for Pb^2+^, Al^3+^, Fe^3+^, Cr^3+^, Pd^2+^, and Au^3+^. Moreover, CD-micelles exhibited ABTS•^+^ radical scavenging ability and cytotoxicity for cisplatin-resistant lung cancer cells. CD-micelles killed cisplatin-resistant small-cell lung cancer cells in a dose-dependent manner with a cancer cell survival rate down to 12.8 ± 4.2%, with a similar treatment function to that of cisplatin. Consequently, CD-micelles functionalized as novel antioxidants show great potential as anticancer nanodrugs in cancer treatment.

## 1. Introduction

Carbon dots (C-dots), deemed as photoluminescent nanomaterials, have attracted considerable attention from scientists because of their unique photophysical properties, biocompatibility, environmental friendliness, easy storage, and low cost [1,2,3]. C-dots can be synthesized through hydrothermal treatment, thermal decomposition, electrochemical oxidation, or ultrasonic synthesis, with advantages of being easy to produce, photophysical stability, and eco-efficiencies [4,5].

C-dots have been applied in sensors, electronic devices, adsorbents, energy storage, catalysis, cell imaging, and cancer therapy [6,7,8,9,10,11,12,13]. For sensors, C-dots are used for sensing Fe^3+^, Hg^2+^, Cu^2+^, Co^2+^, Au^3+^, Cr^6+^, ClO^−^, S^2−^, S_2_O_3_^2−^, Pb^2+^, H_2_O_2_, cysteine, glutathione, synthetic cathinones, flunitrazepam, or nimetazepam [14,15,16,17,18] through mechanisms including the inner filter effect and dynamic or static processes [19,20,21]. For adsorbents, Zhang et al. synthesized C-dot-embedded mesoporous silica nanoparticles in 2015, which had high adsorption capacity for Cu^2+^, Pb^2+^, and Hg^2+^ [11]. In 2020, Issa et al. produced environmentally friendly C-dots from tapioca flour, with a removal efficiency of 80.6% for Pb^2+^ [12]. In 2024, Valente et al. synthesized poly(β-cyclodextrin)-modified C-dots, with removal efficiencies of almost 100% for Ni^2+^, Cu^2+^, Cd^2+^, and Pb^2+^ [13].

In cancer therapy, C-dots have been used in photothermal therapy, photodynamic therapy, and drug delivery [22,23]. Among different types of cancer, lung cancer has the fastest growing rates of morbidity and mortality [24,25]. The effectiveness of chemotherapy is frequently hampered by the low therapeutic index of drugs and the occurrence of inherent and acquired drug resistance in cancer cells [26,27]. Zhao et al. synthesized C-dots loaded with cisplatin(IV) prodrug and doxorubicin to monitor the location of nanocarriers and drug release; they exhibited anticancer effects for the effective apoptosis of A2780 and A2780cis cancer cells with cisplatin resistance [28].

C-dot precursors, such as fruits, food, beverages, vegetables, leaves, and waste materials, have been widely studied [11,29]. Leek seeds deeded as biomaterials contain oil, crude proteins, and dietary fibers; these seeds have several biological effects, including anticancer, antimicrobial, and antioxidant effects, and they are used in traditional Chinese medicine [30,31,32]. However, leek seeds have not been studied as a precursor of C-dots. 

Thus, leek seeds were selected in this study as starting materials because of their significant anticancer, antimicrobial, and antioxidant activities [30,31,32]. Leek seed extract was obtained through a supercritical fluid extraction process and then sequentially carbonized using a dry heating method, followed by hydrolysis to form C-dot micelles (CD-micelles). The results show that CD-micelles have multifunctional properties, including applications for Co^2+^ sensors and metal ion removal, as well as for anticancer nanodrugs, providing promising applications for C-dots.

## 2. Materials and Methods

### 2.1. Materials

Lithium chloride (LiCl), silver nitrate (AgNO_3_), zinc nitrate hexahydrate [Zn(NO_3_)_2_·6H_2_O], and potassium persulfate (K_2_S_2_O_8_) were purchased from Acros Organics (Geel, Antwerp, Belgium). Chromium(III) nitrate nonahydrate [Cr(NO_3_)_3_·9H_2_O], hexadecyltrimethylammonium bromide (CTAB), sodium dodecyl sulfate (SDS), fluorescein, ethyl acetate, and ethanol were purchased from Fisher Chemical (Fisher Scientific, Waltham, MA, USA). Palladium chloride (PdCl_2_), gold chloride trihydrate (HAuCl_4_·3H_2_O), and 2,2′-Azino-bis(3-ethylbenzothiazoline-6-sulfonic acid) (ABTS) were purchased from Combi-Blocks (San Diego, CA, USA). Cis-diamminedichloroplatinum(II) (cisplatin) was obtained from AK Scientific (Union, CA, USA). Sodium hydroxide (NaOH), mercuric nitrate dihydrate [Hg(NO_3_)_2_·2H_2_O], cupric nitrate trihydrate [Cu(NO_3_)_2_·3H_2_O], ferric nitrate nonahydrate [Fe(NO_3_)_3_·9H_2_O], aluminum nitrate nonahydrate [Al(NO_3_)_3_·9H_2_O], lead nitrate [Pb(NO_3_)_2_], nickel nitrate hexahydrate [Ni(NO_3_)_2_·6H_2_O], and cobalt nitrate hexahydrate [Co(NO_3_)_2_·6H_2_O] were purchased from Echo Chemical (Toufen, Miaoli, Taiwan). Deuterated oxide (D_2_O) was purchased from Acros Organics (Geel, Antwerp, Belgium). Carbon dioxide (purity 99.99%) was supplied by San Ying Gas Co., LTD. (New Taipei City, Taiwan). Leek seeds were obtained from a Chinese herb medicine store (Chiayi County, Taiwan). Ultrapure water (18.2 mΩ·cm) was obtained using an ultrapure water system (Direct-Q3; Merck Millipore, Billerica, MA, USA) and used to prepare all of the aqueous solutions in this study.

### 2.2. Leek Seed Extract

The leek seeds were ground in a knife mill (CS-700; Cosuai, Jinhua, China) and then filtered through a sieve with a diameter range of 0.150–0.850 mm to yield leek seed powder, which was divided into samples of 600 g each. The leek seed powder was then extracted using supercritical carbon dioxide (scCO_2_) at 333 K and 5000 psi with a continuous flow rate of 6 L·min^−1^ for 8 h. The extraction was performed using supercritical fluid extraction equipment (OV-SCF-10000 Series; Taiwan Supercritical Technology, Changhua, Taiwan) equipped with an extractor with a volume of 1000 cm^3^. Light-yellow leek seed extract was obtained and then stored in a refrigerator at 4 °C until further use. The weight of the obtained seed extract was 16.6 g.

### 2.3. Analysis of Leek Seed Extract Through Gas Chromatography–Mass Spectrometry

The leek seed extract (50 μL) was dissolved in 500 μL of ethanol for gas chromatography–mass spectrometry (GC–MS) analysis (Agilent 8890/5977B, Santa Clara, CA, USA) on a fused-silica capillary column (DB-5MS; 30 m × 0.32 mm i.d.; film thickness: 0.25 µm; J&W Scientific, Koeniz, Switzerland). The parameters of the GC–MS system were as follows: electron impact mode, 70 eV; carrier gas, helium; carrier gas flow rate, 1.0 mL⋅min^−1^; and injection port temperature, 260 °C. The temperature was initially maintained at 135 °C for 0.5 min and then gradually increased to 300 °C at a rate of 70 °C min^−1^ and maintained at 300 °C for 12.5 min.

### 2.4. Synthesis of CD-Micelles

The leek seed extract (5 g) was added to a Teflon-lined stainless steel autoclave and heated in a furnace at 200 °C for 3 days. After cooling to ambient temperature, a brown-yellow mixture was obtained. Subsequently, to form CD-micelles, an aliquot (1.0 g) of the obtained mixture was reacted with 5 mL of NaOH solution (0.02 N) in an ultrasonic bath at 60 °C for 120 min. To remove organic impurities, liquid–liquid extraction was performed with a solution of ethyl acetate and ultrapure water (10 mL, *v*/*v* = 1/1). The purified CD-micelles were freeze-dried. The stock aqueous solution of the purified CD-micelles was prepared at a concentration of 16 mg mL^−1^ and stored at 4 °C before use.

### 2.5. Characterization of CD-Micelles

The absorption spectra of CD-micelles were obtained using an ultraviolet-visible (UV-Vis) spectrophotometer (Evolution 220; Thermo Fisher Scientific, Waltham, MA, USA). The photoluminescence (PL) spectra of CD-micelles were recorded using a microplate reader (SpectraMax i3x; Molecular Devices, San Jose, CA, USA). The PL quantum yield (Φ_PL_) of CD-micelles was estimated by comparing their PL intensity at 460 nm (excited at 365 nm) and absorbance at 365 nm with those of quinine sulfate dissolved in 0.1 M H_2_SO_4_ (Φ_PL_ = 0.54). The absorbance values of CD-micelles and quinine sulfate were kept under 0.1 at their excitation wavelength to minimize the re-absorption effect. The Fourier-transform infrared (FTIR) spectra of CD-micelles were recorded using a spectrometer (Nicolet iS5; Thermo Scientific, Waltham, MA, USA). The sizes and shapes of CD-micelles with and without Co^2+^ were recorded using a high-resolution transmission electron microscope (HRTEM) (JEM-2100F; JEOL, Tokyo, Japan) coupled with an energy dispersive X-ray spectrometer at 200 kV. Surface elements and bonding states of CD-micelles were investigated using an X-ray photoelectron spectroscopy (XPS) instrument from VG Scientific (East Grinstead, UK) with Al Kα X-ray radiation. Dynamic light scattering (DLS) measurements (Nano-ZS90; Malvern Panalytical, Malvern, UK) were employed to measure the average sizes of CD-micelles dispersed in ultrapure water. A nuclear magnetic resonance (NMR) instrument (Ultrashield-400, Bruker, Billerica, MA, USA) was used to perform ^1^H NMR and ^13^C NMR analyses.

Fluorescein was used to evaluate the critical micelle concentration (CMC) of CD-micelles. Briefly, 8 aliquots (125 µL) of fluorescein aqueous solution (0.32 µM) were separately mixed with 125 µL of CD-micelle solutions with concentrations from 0 to 0.06 mg mL^−1^ before being subjected to PL measurements. The CMC value of CD-micelles was assessed based on their PL intensity at 515 nm emitted from fluorescein under an excitation wavelength of 460 nm as concentrations of CD-micelles were increased. 

### 2.6. Selectivity and Sensitivity of CD-Micelles for Metal Ions

To assess the selectivity of CD-micelles for metal ions, 150 μL aliquots of the CD-micelle solution (3.2 mg mL^−1^) were separately mixed with 150 μL of metal ion solutions (200 μM), namely Li^+^, Zn^2+^, Al^3+^, Pb^2+^, Fe^3+^, Cu^2+^, Ni^2+^, Co^2+^, Ag^+^, Cr^3+^, Pd^2+^, and Au^3+^. The PL spectra of CD-micelles with and without metal ions were separately recorded at an excitation wavelength of 365 nm. The PL intensities of CD-micelle solutions at 450 nm were separately plotted against the tested metal ions. In addition, the sensitivity of CD-micelles for Co^2+^ was assessed. Aliquots (150 μL) of the CD-micelle solution (3.2 mg mL^−1^) were separately mixed with 150 μL of Co^2+^ solutions of various concentrations (0–80 μM). Subsequently, the PL spectra of CD-micelles with and without Co^2+^ were separately recorded. The PL quenching efficiency at 450 nm of the CD-micelles against Co^2+^ at concentrations of 1–40 μM was thus used to evaluate the sensitivity. The PL quenching behavior between CD-micelles and Co^2+^ was assessed by using the Stern–Volmer equation shown in Equation (1).

F_0_/F = 1 + Ksv [Q] (1)

where F_0_ and F are the PL intensities of CD-micelles in the absence and presence of the quencher, respectively. [Q] is the concentration of Co^2+^, and K_SV_ is the corresponding Stern–Volmer constant. In addition, the lifetimes of CD-micelles with and without Co^2+^ (100 μM) were measured using a steady-state PL spectrometer with a picosecond pulsed LED (Ex 313 nm) (FS5; Edinburgh Instruments, Livingston, UK) at an emission wavelength of 450 nm. All experiments were performed under ambient pressure and temperature conditions.

### 2.7. Metal Ion Removal Using CD-Micelles Through Liquid–Liquid Extraction

Aliquots (2 mL) of the CD-micelle solution (16 mg mL^−1^) were separately mixed with metal ion solutions (2 mL, 500 µM) containing Li^+^, Zn^2+^, Ni^2+^, Fe^3+^, Pb^2+^, Cu^2+^, Co^2+^, Ag^+^, Al^3+^, Cr^3+^, Pd^2+^, or Au^3+^. Subsequently, to adjust the pH to 4 and 7, 0.2 M HNO_3_ was separately added into the mixtures. Ethyl acetate (4 mL) was added to each mixture and stirred for 2 h. The organic and aqueous layers were separated, and the organic layer was removed. Aliquots (100 µL) of the remaining solutions were separately diluted in 1 mL of ultrapure water for analysis. Before and after liquid–liquid extraction, the metal ion concentrations of the remaining solutions were determined using inductively coupled plasma–optical emission spectroscopy (ICP-OES, PerkinElmer Optima 8300, Waltham, MA, USA). The efficiency of metal ion removal was calculated using Equation (2).

Removal efficiency (%) = [M]_a_/[M]_b_ × 100 (%) (2)

where [M]_a_ and [M]_b_ represent the metal ion concentrations after and before liquid–liquid extraction, respectively. For comparison, SDS and CTAB were employed with similar metal ion removal processes for CD-micelles.

### 2.8. Antioxidant Activity of CD-Micelles

The CD-micelle stock solution (16 mg mL^−1^) was separately diluted in ultrapure water for the preparation of diluted CD-micelle solutions of various concentrations (5, 25, 50, 100, 500, and 1000 μg mL^−1^). Subsequently, 1 mL aliquots of the as-diluted CD-micelle solutions/ultrapure water were mixed with 1 mL of ABTS (100 µM). The reaction was allowed to proceed for 2 h in the dark. Subsequently, the absorption spectra of these mixtures were recorded. The radical scavenging efficiency of CD-micelles was calculated using Equation (3) [33,34].

Radical scavenging efficiency (%) = (Ar − As)/Ar × 100 (%)(3)

where Ar is the absorbance at 734 nm of ABTS•^+^ in ultrapure water and As is the absorbance at 734 nm for ABTS•^+^ with CD-micelles.

### 2.9. Cytotoxicity of CD-Micelles Against Cisplatin-Resistant Lung Cancer Cells

The potential of CD-micelles to overcome drug (cisplatin) resistance was assessed using an established cisplatin-resistant lung cancer model [35,36]. Small-cell lung cancer (SCLC) cells and related SCLC-cisplatin-resistant (SCLC-cisplatin^R^) cells were grown in Dulbecco’s modified Eagle’s medium supplemented with 10% fetal bovine serum at 37 °C in a 5% CO_2_ atmosphere. SCLC-cisplatin^R^ cells were cultured with 0.15 μM cisplatin (Sigma-Aldrich, St. Louis, MA, USA) to maintain the resistant phenotypes that were provided from the M.D. Anderson Cancer Center [37,38]. The cytotoxicity of cisplatin and CD-micelles against SCLC-cisplatin^R^ cells was determined using MTT assays. Briefly, SCLC-cisplatin^R^ cells were seeded in 96-well plates at a density of 3 × 10^3^ cells per well. Subsequently, SCLC-cisplatin^R^ cells in plates were separately treated with increasing doses of cisplatin (0.02–9.6 μM) and CD-micelles (1.4–72.3 μg mL^−1^) for 72 h, respectively [38,39]. The MTT reagent was then added at a concentration of 5 mg mL^−1^ for 2 h. Finally, the medium was replaced with 200 μL of DMSO to dissolve the crystal violet precipitate. The optical density of each well at 490 nm was measured using a microplate reader (Thermo Scientific, Waltham, MA, USA).

## 3. Results and Discussion

### 3.1. Characterization of Leek Seed Extract and CD-Micelles

**Scheme 1:**
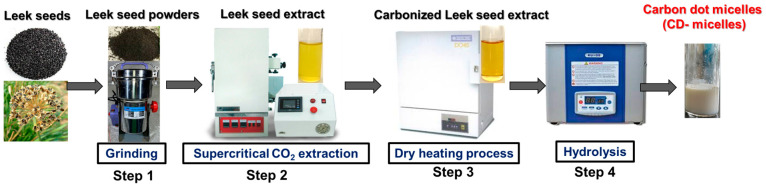
The process of the synthesis of CD-micelles from leek seeds.

Scheme 1 illustrates the synthesis of CD-micelles from leek seeds. In step 1, leek seeds were ground using a knife mill and then filtered through a sieve with a diameter range of 0.150–0.850 mm to yield leek seed powders. In step 2, scCO_2_ extraction was employed, with the advantages of high extraction yield and environmental friendliness [40,41]. The leek seed extract (yield: 2.8%) was obtained after performing scCO_2_ extraction for 8 h. Figure S1 presents the total ion chromatograph of the leek seed extract, which was obtained from GC–MS analysis. The identified components are summarized in Table 1. Ten components were detected in the leek seed extract: n-hexadecanoic acid, 9(Z),12(Z)-octadecadienoic acid, octadecanoic acid, (Z, E)-7,11-hexadecadien-1-yl acetate, eicosanoic acid, butyl 9,12-octadecadienoate, palmitin [hexadecanoic acid, 2-hydroxy-1-(hydroxymethyl)ethyl ester], linolein [9,12-octadecadienoic acid (Z, Z)-, 2-hydroxy-1-(hydroxymethyl)ethyl ester], squalene, and cholesterol. The MS spectra are shown in Supporting Information I. Of these components, n-hexadecanoic acid, octadecanoic acid, and linolein were the three major ingredients [42].

**Figure 1:**
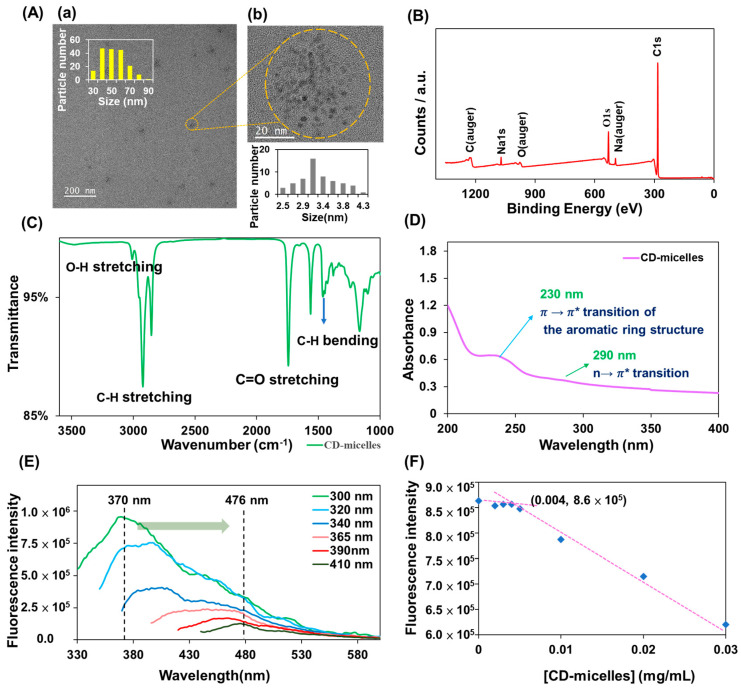
(**A**) HRTEM images, (**B**) XPS spectrum, and (**C**) FTIR spectrum of CD-micelles. (**D**) Absorption spectrum of CD-micelles in ultrapure water (0.16 mg mL^−1^) and (**E**) PL spectra of CD-micelles when excited at wavelengths of 300 to 410 nm. (**F**) Critical micelle concentration (CMC) assessment of CD-micelles. The insets (**a**,**b**) in (**A**) represent an enlarged image of the CD-micelles and their size distribution, respectively.

The leek seed extract was carbonized through hydrolysis, polymerization, carbonization, and passivation using a dry heating method (step 3), followed by hydrolysis (step 4) to form CD-micelles [43,44]. The average diameter of the CD-micelles was 3.3 ± 0.4 nm (calculated from 50 counts) (Figure 1A(a)), which is similar to previous findings for C-dots [5]. Their micelles had an average diameter of 56.9 ± 14.1 nm (from 180 counts) in HRTEM images (Figure 1A(b)). In addition, the average size of the CD-micelles in ultrapure water was 178.4 nm based on the DLS analysis. Figure 1B represents the XPS spectrum of the CD-micelles; the data demonstrated three peaks obtained at 284, 532, and 1071 eV, which corresponded to C 1s, O 1s, and Na 1s, respectively [13,15,45]. Figure S2A depicts the deconvoluted C 1s peaks obtained at 284.6, 286.0, and 288.7 eV, which corresponded to C-C, C-O, and C=O, respectively [13,15]. The deconvoluted XPS spectrum of O 1s revealed two peaks at 532.5 and 531.9 eV, which were attributed to C-O and C=O, respectively (Figure S2B) [13,15]. The Na 1s signal was detected in the spectrum primarily because CD-micelles were formed through hydrolysis with NaOH. Thus, Na^+^ absorbed into the functional groups of CD-micelles resulted in the charge neutrality of the micelles. Figure 1C depicts the FTIR spectrum of the CD-micelles, showing a weaker and broader O–H stretching band at 3475 cm^−1^; moderate C–H stretching bands at 3009, 2918, and 2854 cm^−1^; a sharp C=O stretching band at 1737; a sharp (asymmetric) and a weak (symmetric) COO^−1^ stretching band at 1557 and 1378 cm^−1^, respectively; a moderate C–H stretching band at 1461 cm^−1^; and a strong C–O stretching band at 1163 cm^−1^ [46,47,48]. The ^1^H NMR spectrum (400 MHz; D_2_O; ppm) of the CD-micelles, as shown in Figure S3, reveals that signals at around 8.37, 3.58~3.48, 2.10, 1.83, 1.51,1.23, and 0.81 are obtained, which are mainly attributed to aldehyde, ester/ether, and alkenyl groups on the surface [49]. As for weak ^1^H signals at around 6.76~6.44, they may correspond to aromatic groups on the surface of CD-micelles [49]. In addition, the ^13^C NMR spectrum of the CD-micelles was provided in Figure S4, showing aldehyde carbon atoms (171), aromatic ester/ether/alcohol carbon atoms (115, 72~68, 62), and aliphatic carbon atoms (37~21, 14, and 13 ppm) [50]. The ^1^H and ^13^C NMR spectra of CD-micelles are similar to those of C-dots in the literature [51], and it is noted that ^1^H signals of alcohol groups on CD-micelles are not observed because of those protons undergoing fast exchanges with D_2_O solvent [52]. The absorption spectrum of the CD-micelles in ultrapure water (0.16 mg mL^−1^) revealed a broad band at 230 nm and a tail from 265 to 350 nm, corresponding to π−π* and n−π* transitions, respectively (Figure 1D) [53,54]. The excitation-wavelength-dependent PL property of the CD-micelles excited at the wavelengths of 300 to 410 nm is illustrated in Figure 1E. This property is attributed to the transitions of the nonbonding orbitals of CD-micelles into their π* orbitals [53,54,55]. Because the aggregated stacking of CD-micelles induced PL quenching, their Φ_PL_ was estimated to be <1%, which was mainly attributed to J-type aggregation [56,57,58].

Figure 1F shows the CMC assessment for CD-micelles by PL intensities at 515 nm of fluorescein against CD-micelle concentrations (0–0.03 mg mL^−1^); a cross point at the concentration of CD-micelle (0.004 mg mL^−1^) was obtained. As CD-micelle concentrations increased over 0.004 mg mL^−1^, the PL intensities of fluorescein decreased significantly. This is attributed to the fact that as the as-prepared C-dots started to form CD-micelles, PL scattering occurred [59].

### 3.2. Detection of Metal Ions Using CD-Micelles

**Figure 2:**
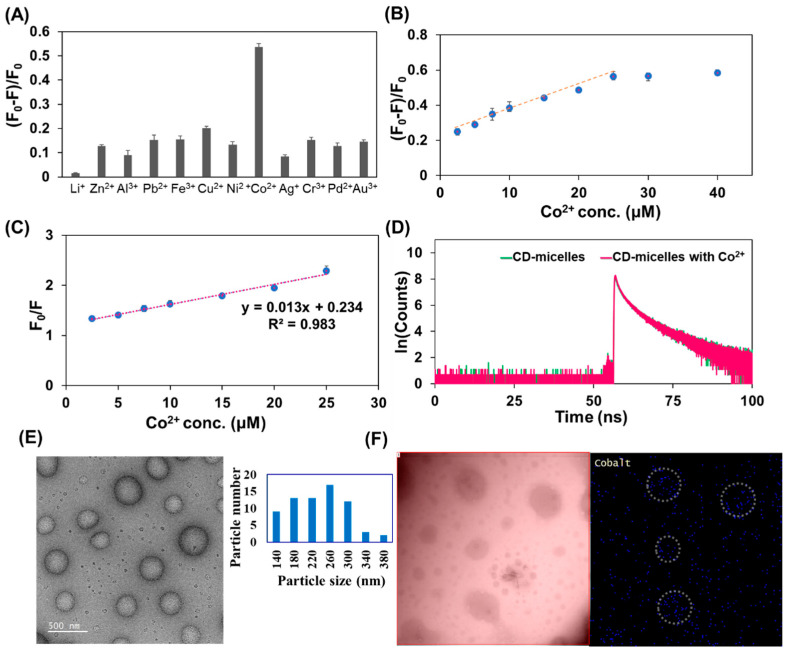
(**A**) PL quenching efficiencies of CD-micelles (1.6 mg mL^−1^) in the presence of various metal ions (100 μM). (**B**) PL quenching efficiencies of CD-micelles against Co^2+^ (0–40 μM). (**C**) The Stern–Volmer plot of CD-micelles against Co^2+^. (**D**) Fluorescence lifetime decay curves of CD-micelles with and without Co^2+^. (**E**) The HRTEM images of CD-micelles with Co^2+^ and their size distribution. (**F**) Energy dispersive X-ray spectrometry mapping images of CD-micelles with Co^2+^.

Figure 2A presents the PL quenching efficiencies of CD-micelles (1.6 mg mL^−1^) in the presence of various metal ions (100 μM), namely Li^+^, Zn^2+^, Ni^2+^, Fe^3+^, Pb^2+^, Cu^2+^, Co^2+^, Ag^+^, Al^3+^, Cr^3+^, Pd^2+^, and Au^3+^. The ion most effectively quenched was the Co^2+^, indicating that CD-micelles have high selectivity for Co^2+^. Figure 2B depicts the PL quenching efficiencies of CD-micelles against Co^2+^ (0–40 μM). A dynamic range from 2.5 to 25 μM was obtained, and the limit of detection was 1.7 μM, calculated using the equation 3σ/m, where σ is the standard deviation of the blank signal (*n* = 3) and m is the slope of the linear range. To examine the Co^2+^ quenching behavior of the CD-micelles, a Stern–Volmer plot was employed. A linear relationship between the Co^2+^ concentration (2.5–25 μM) and its Stern–Volmer constant of 1.3 × 10^−2^ μM^−1^ was observed (Figure 2C) [17]. A linear Stern–Volmer plot indicates that only dynamic or static quenching occurred between CD-micelles and Co^2+^ [17]. In this study, to confirm the quenching mechanism, fluorescence lifetime decay curves of CD-micelles with and without Co^2+^ were measured (Figure 2D). The two curves exhibited adequate overlap. In addition, the average lifetimes of CD-micelles with and without Co^2+^ were calculated to be 8.2 ns and 8.1 ns, respectively. Because no significant difference in the lifetimes of CD-micelles with and without Co^2+^ was observed, the dynamic quenching mechanism was ruled out [60]. Moreover, Figure 2E shows an unexpected HRTEM image of CD-micelles with Co^2+^, with an average diameter of 240.3 ± 66.9 nm (from 70 counts). To prove that Co^2+^ was present in these particles, energy dispersive X-ray spectrometry was conducted (Figure 2F). The results revealed the presence of Co^2+^ in the particles, with a weight percentage of 1.18%. These results confirmed that the Co^2+^ quenching behavior of the CD-micelles was a static process [17,60]. Additionally, CD-micelles interacted with Co^2+^ to form new spherical particles [61,62,63]. The detailed mechanisms of CD-micelles with high selectivity towards Co^2+^ and their formation of the spherical particles are unclear, but it is supposed that functional groups on the surface of CD-micelles, including O–H, C=O, and COO^−1^, have special interactions with Co^2+^ [64]. To confirm this conjecture, ^1^H NMR spectra of CD-micelles without and with Co^2+^ presented in Figure S5 show that the ^1^H peak of aldehyde groups on the surface of CD-micelles shifted slightly from 8.37 to 8.40 when Co^2+^ existed, indicating that aldehyde groups on CD-micelles have certain interactions with Co^2+^. On the other hand, Co^2+^ induced ^1^H peaks of CD-micelles to be broader ones due to their paramagnetic properties [65]. Unfortunately, because of fast exchanges of hydrogen in alcohol groups on CD-micelles with D_2_O, it is difficult to monitor their O–H chemical shifts for CD-micelles without and with Co^2+^ in aqueous environments using NMR measurements [52]. Lastly, carbon dots using natural biomaterials were selected for comparison. Their produced methods, precursors, and corresponding LODs are summarized in Table 2 [66,67,68]. A report involving carbon dots using straws as precursors through a hydrothermal method showed PL quenching against Co^2+^ with an LOD of 0.38 μM based on a static quenching mechanism and the inner filter effect [66]. In addition, limes and kelps were used to produce carbon dots through a microwave method. These two carbon dots showed PL quenching towards Co^2+^, with LODs of 0.39 and 1.63 μM, based on the inner filter effect and the electron transfer mechanism, respectively [67,68].

### 3.3. CD-Micelles for Metal Ion Removal Through Liquid–Liquid Extraction

**Figure 3:**
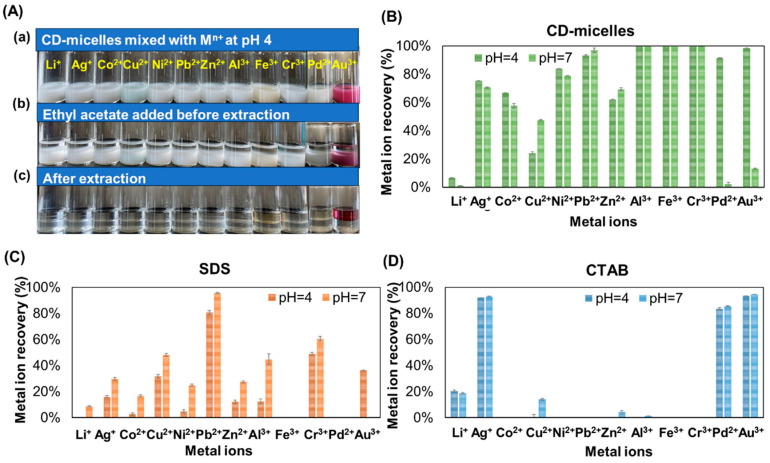
(**A**) CD-micelles employed for metal ion removal through liquid–liquid extraction by using ethyl acetate. Removal efficiencies of (**B**) CD-micelles, (**C**) SDS, and (**D**) CTAB for various metal ions.

**Figure 4:**
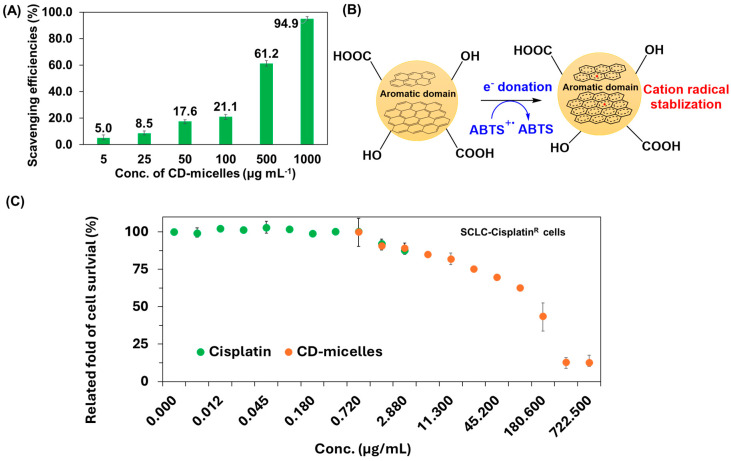
(**A**) CD-micelles for ABTS•^+^ scavenging efficiencies. (**B**) Illustrated mechanism for CD-micelles reacted with ABTS•^+^. (**C**) The cell survival ratios of SCLC-cisplatin^R^ cells against cisplatin and CD-micelles.

**Scheme 2:**
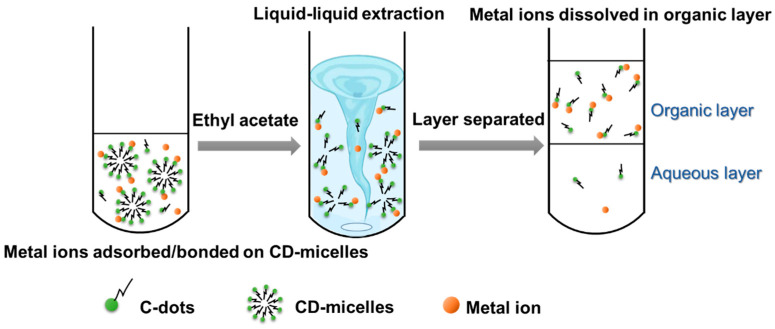
A proposed mechanism for CD-micelles to remove metal ions through liquid–liquid extraction.

Figure 3A presents the process through which CD-micelles were functionalized as adsorbents and chelating agents to employ metal ion removal through liquid–liquid extraction. As depicted in Figure 3A(a), CD-micelle solutions were separately mixed with metal ion solution that contained Li^+^, Ag^+^, Co^2+^, Cu^2+^, Ni^2+^, Pb^2+^, Zn^2+^, Al^3+^, Fe^3+^, and Cr^3+^ sequentially. After adjustment of the pH value of the above mixtures to 4 or 7 by using 0.2 M HNO_3_, ethyl acetate was added to each mixture (Figure 3A(b)). Liquid–liquid extraction was performed, and then we waited for solvent separation (Figure 3A(c)). The removal efficiencies of CD-micelles against various metal ions at pH 4 and 7 are provided in Figure 4B. The results indicated that through liquid–liquid extraction in acidic conditions (pH 4), CD-micelles extracted Pb^2+^, Al^3+^, Fe^3+^, Cr^3+^, Pd^2+^, and Au^3+^ with efficiencies of >90%. Moderate removal efficiencies (40–80%) were obtained for CD-micelles against Ag^+^, Co^2+^, Cu^2+^, Ni^2+^, and Zn^2+^, and low removal efficiency (7%) was obtained for Li^+^. This low removal efficiency indicated the poor interaction between CD-micelles and Li^+^, which is mainly attributed to the low positive charge (I) and small size of Li^+^. In neutral conditions (pH 7), CD-micelles show similar ion-extracted properties, except for Pd^2+^ and Au^3+^. For comparison, ion removal efficiencies for Pb^2+^ using SDS and those for Ag^+^, Pd^2+^, and Au^3+^ using CTAB of over 80% were obtained. The sulfate group in SDS had a strong interaction with Pb^2+^ to form an SDS–Pb^2+^ complex [69,70,71,72]. Similarly, Ag^+^, Pd^2+^, and Au^3+^ interacted with CTAB to form complexes [73,74,75], and those complexes were thus extracted through liquid–liquid extraction. Regarding metal ion removal using CD-micelles through liquid–liquid extraction, the illustrated mechanism is depicted in Scheme 2. Metal ions may be absorbed/bonded on the functional groups in CD-micelles. Subsequently, these metal ion absorbed/bonded CD-micelles disintegrate and are then dispersed in ethyl acetate during liquid–liquid extraction. After the separation of the organic and aqueous layers, metal ions are transferred in the ethyl acetate layer through absorption/bonding onto C-dots.

### 3.4. Antioxidant and Anticancer Activities of CD-Micelles

CD-micelles for ABTS•^+^ scavenging efficiency obtained from the colorimetric assay are presented in Figure 4A. The CD-micelles exhibited dose-dependent scavenging efficiencies ranging from 5.0% to 94.9% at concentrations ranging from 5 to 1000 μg mL^−1^ [76,77]. As depicted in Figure 4B, CD-micelles exhibited an ABTS•^+^ scavenging ability to form ABTS, which is mainly attributed to their π-conjugated bonds on the surfaces that stabilized the cation radical, which indicates that CD-micelles are functionalized as antioxidants [76,77]. The cytotoxicity of cisplatin and CD-micelles was evaluated using the MTT assay, as shown in Figure 4C. The results showed a small reduction in the survival rate of SCLC-cisplatin^R^ cells from 99.2 ± 2.4% to 87.7 ± 0.5%, even at the highest concentration of cisplatin (2.88 μg mL^−1^ or 9.60 μM), which indicates the occurrence of cisplatin resistance. In contrast, CD-micelles were administered at concentrations ranging from 1.4 to 723 μg mL^−1^, resulting in a dose-dependent decrease in cell viability, with survival rates decreasing from 90.7 ± 3.2% to 12.8 ± 4.2%. Notably, the cytotoxic effects of CD-micelles at concentrations of 0.72, 1.44, and 2.88 μg mL^−1^ were comparable to those of cisplatin at the same concentrations. These findings suggest that CD-micelles exhibit similar therapeutic efficacy to cisplatin against SCLC-cisplatin^R^ cells. Therefore, CD-micelles could potentially serve as a replacement for cisplatin in cases of cisplatin resistance, thereby reducing the risk of cisplatin overdose and mitigating adverse effects, such as mortality and peripheral neuropathy. Regarding the underlying mechanism, it is unclear, but it is supposed that CD-micelles were functionalized as exogenous antioxidants [78,79]. They probably quenched singlet oxygen (^1^O^−^_2_) anion and peroxyl (•ROO) radicals to remove free radical intermediates or to delay oxidative reactions via several modalities, including alterations in cell signaling, changes in cell cycle progression, and the modulation of enzymatic activities [80,81]. Thus, the results indicated that CD-micelles, as novel antioxidants, exhibit great potential for use in anticancer nanodrugs in cancer therapy [82,83,84].

## 4. Conclusions

In this study, scCO_2_ extraction was used to obtain leek seed extract, which was subsequently carbonized and hydrolyzed to form CD-micelles. The CD-micelles were selective for the quantitation of Co^+2^ through analyte-induced PL quenching, employing a static process with the formation of the self-assembly Co^2+^-CD-micelle sphere particles. In addition, CD-micelles can extract metal ions through liquid–liquid extraction. Removal efficiencies over 90% were obtained using CD-micelles against Pb^2+^, Al^3+^, Fe^3+^, Cr^3+^, Pd^2+^, and Au^3+^. Moreover, CD-micelles functionalized as novel antioxidants killed SCLC-cisplatin^R^ cells in a dose-dependent manner. Thus, CD-micelles exhibit great potential as a replacement for cisplatin when cisplatin resistance occurs, thus preventing cisplatin overdoses in cancer therapy.

## Figures and Tables

**Table 1:** Chemical components of leek seed extract analyzed using GC-MS.

Item	Compound	Retention Time (min)
1	n-Hexadecanoic acid	3.43
2	9(Z),12(Z)-Octadecadienoic acid	3.75
3	Octadecanoic acid	3.78
4	(Z, E)-7,11-Hexadecadien-1-yl acetate	4.14
5	Eicosanoic acid	4.22
6	Butyl 9,12-octadecadienoate	4.53
7	Palmitin [hexadecanoic acid, 2-hydroxy-1-(hydroxymethyl)ethyl ester]	4.65
8	Linolein [9,12-octadecadienoic acid (Z, Z)-, 2-hydroxy-1-(Hydroxymethyl)ethyl ester]	5.31
9	Squalene	5.82
10	Cholesterol	8.23

**Table 2:** Methods, precursors, and LODs of carbon dots produced from natural biomaterials for detection of Co^2+^.

Method	Precursors	Detection Limit (μM)	Reference
Hydrothermal	Straw	0.38	[66]
Microwave	Lime	1.63	[67]
Microwave	Kelp	0.39	[68]
Dry heating	Leek seed extract	1.7	This work

## Data Availability

The datasets used and/or analyzed during this study are available from the corresponding author upon reasonable request.

## Supplementary Materials

The following supporting information can be downloaded at https://www.mdpi.com/article/10.3390/jfb15110347/s1, Figure S1. Total ion chromatograph of the leek seed extract obtained through GC-MS analysis; Figure S2. Deconvoluted (A) C 1s and (B) O 1s XPS spectra of CD-micelles; Supporting Information I: The GC-Electron impact spectra of 10 components obtained from leek seed extract; Figure S3. ^1^H NMR spectrum of the CD-micelles; Figure S4. ^13^C NMR spectrum of the CD-micelles; Figure S5. ^1^H NMR spectra of the CD-micelles without (down) and with Co^2+^ (up).
