# Supplementary material for: Carbon Dot Micelles Synthesized from Leek Seeds in Applications for Cobalt (II) Sensing, Metal Ion Removal, and Cancer Therapy
Source: J Funct Biomater. 2024 Nov 15;15(11):347. doi: 10.3390/jfb15110347 (PMC11595631; doi:10.3390/jfb15110347)
Supplement: Supplementary file 1 [file jfb-15-00347-s001.zip › jfb-3238142-supplementary.pdf]

Journal of
Functional
Biomaterials

MDPI

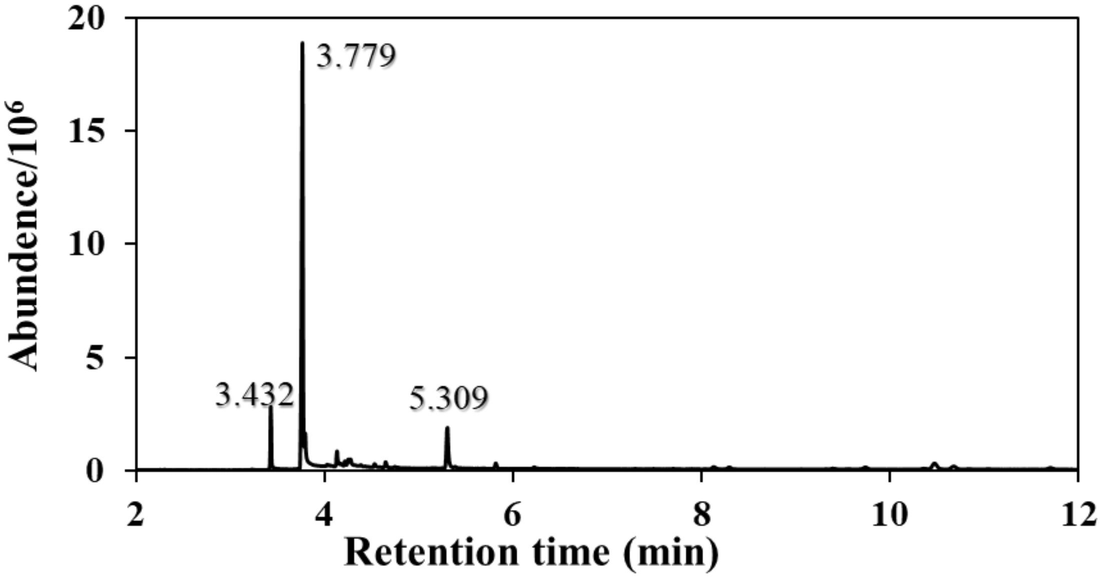

Figure S1. Total ion chromatograph of the leek seed extract obtained from GC-MS analysis. Retention time (Rt) at 3.43 min: n-hexadecanoic acid, Rt at 2.78 min: octadecanoic acid, and Rt at 5.31 min: linolein.

Scheme 10. components obtained from leek seed extract.

1. **Mass spectrum matched with n-hexadecanoic.**

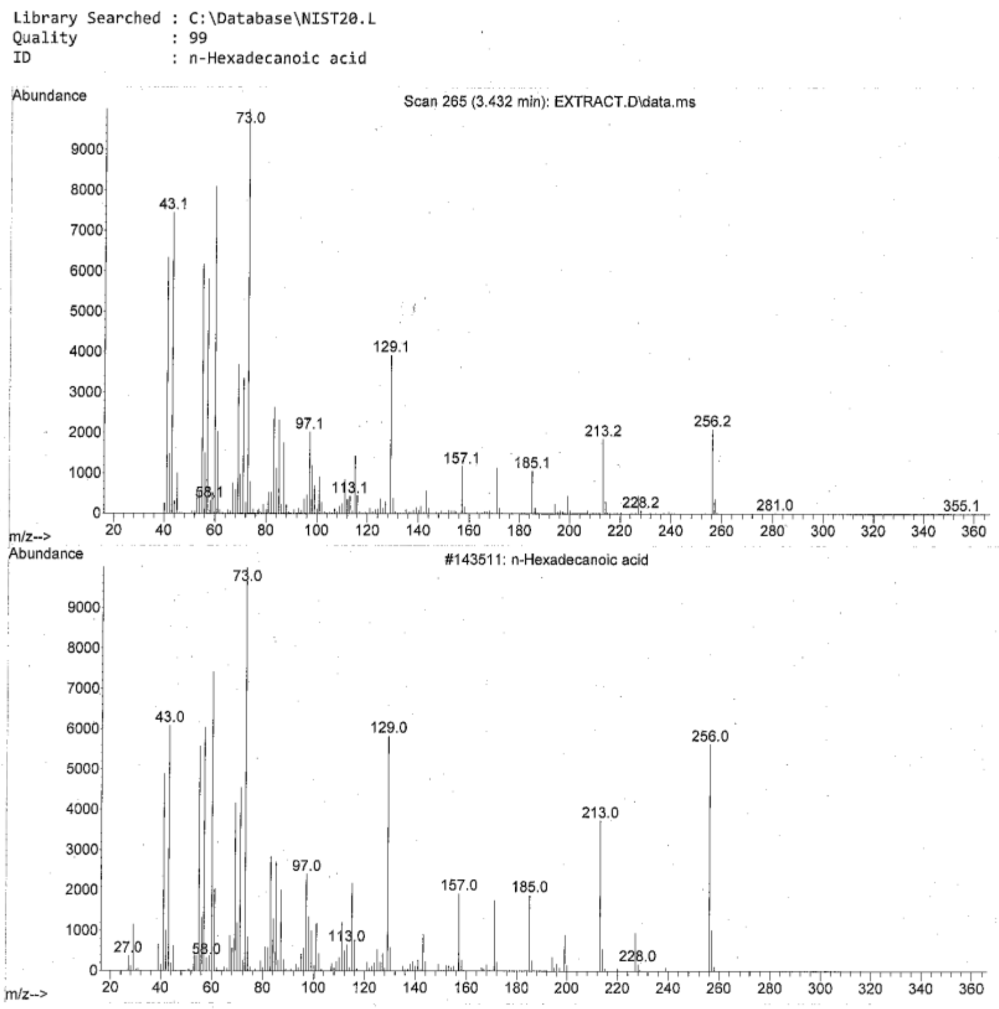

2. Mass spectrum matched with 9(Z),12(Z)-octadecadienoic acid.

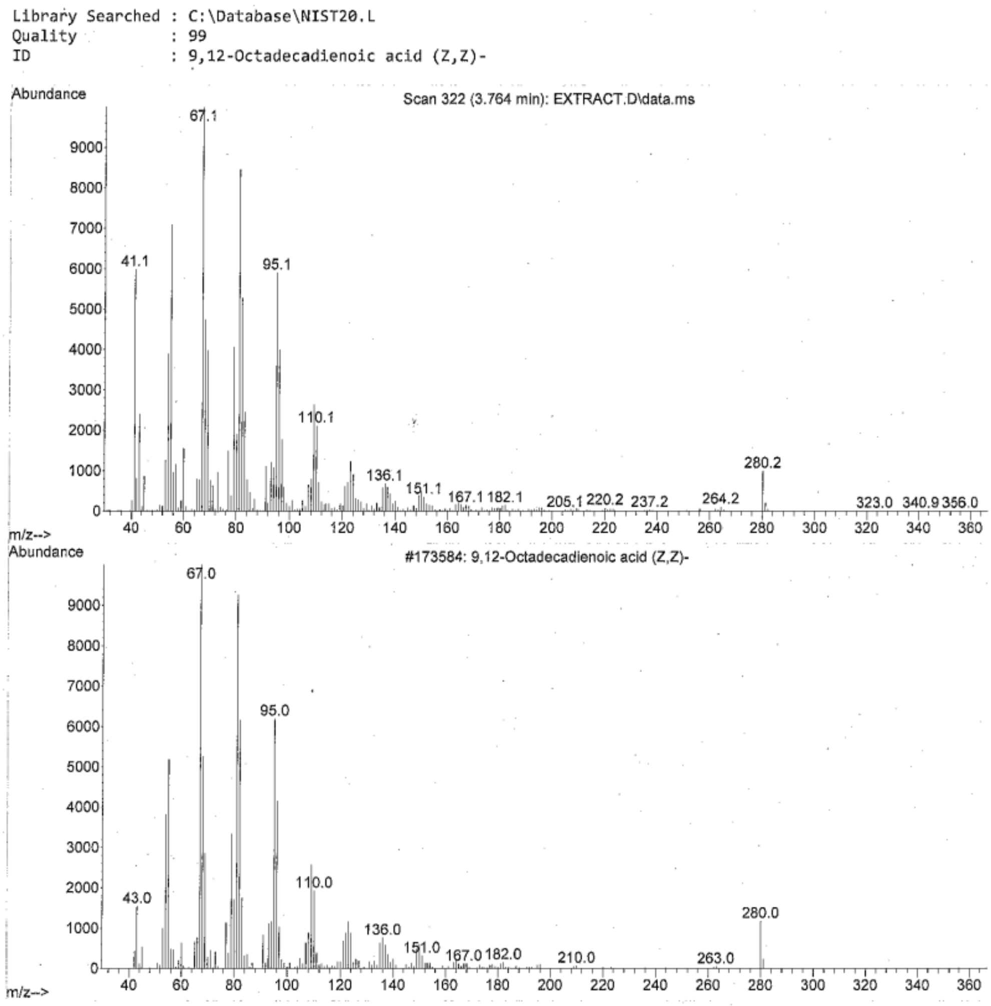

3. Mass spectrum matched with octadecanoic acid.

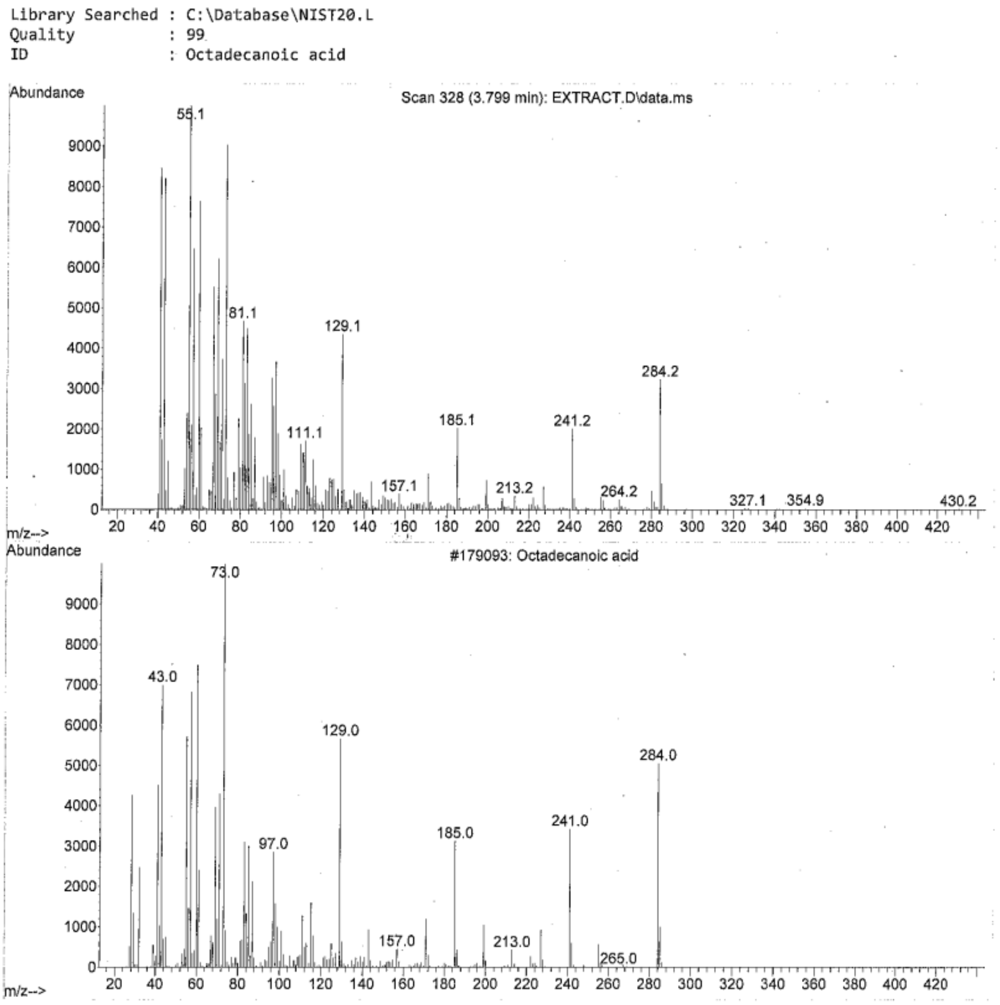

4. Mass spectrum matched with (Z,E)-7,11-hexadecadien-1-yl acetate.

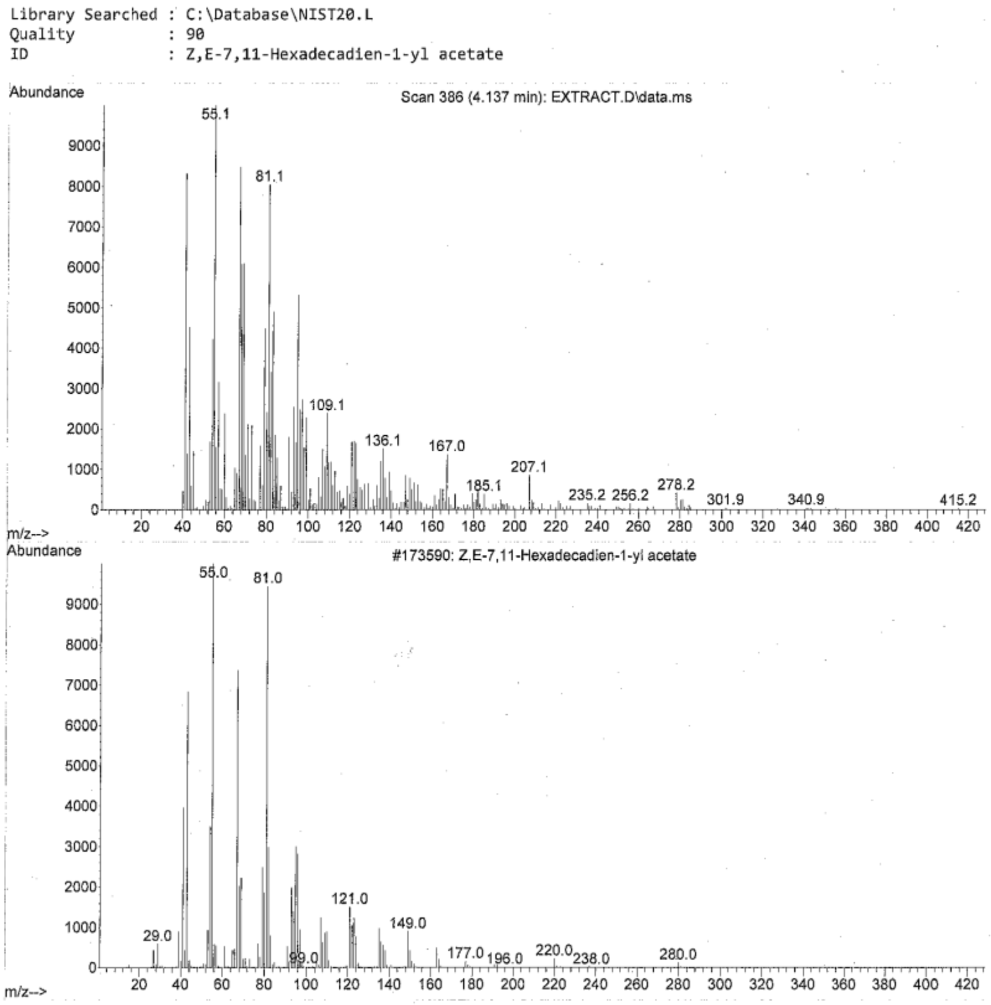

5. Mass spectrum matched with eicosanoic acid.

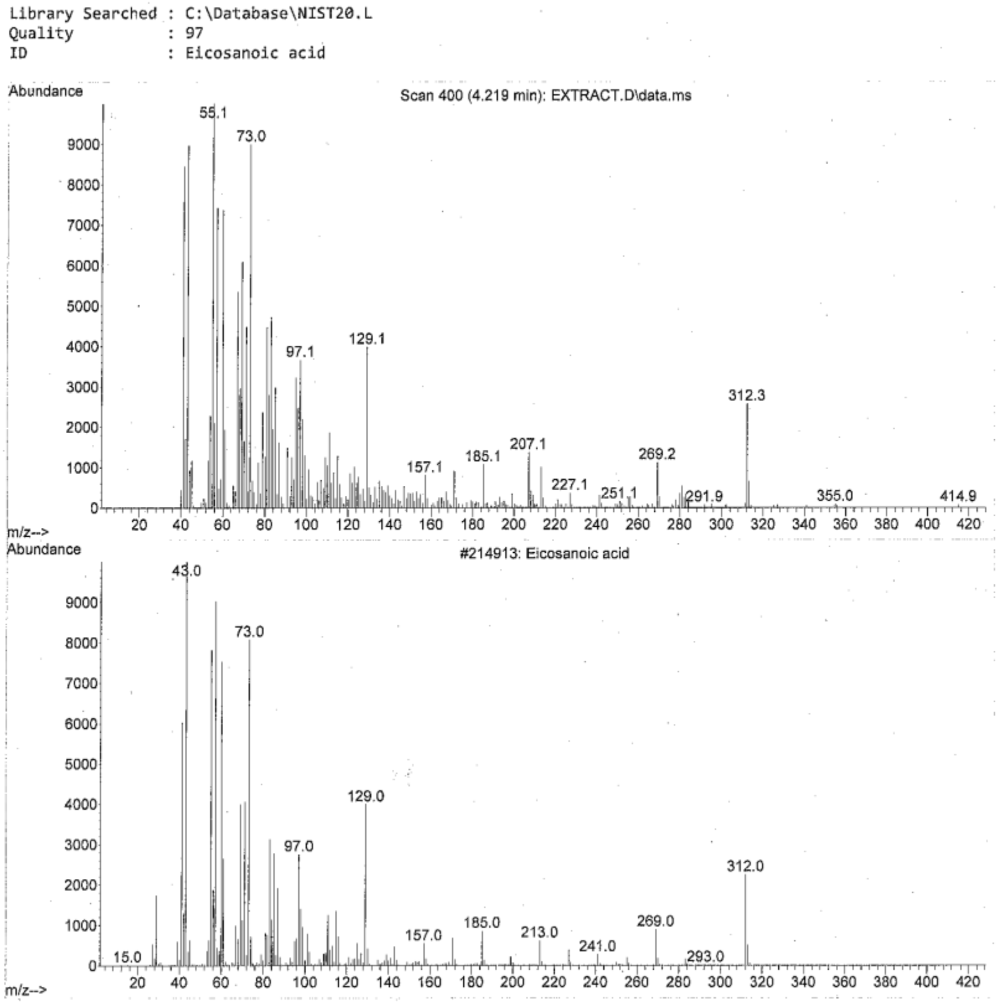

6. Mass spectrum matched with butyl 9,12-octadecadienoate.

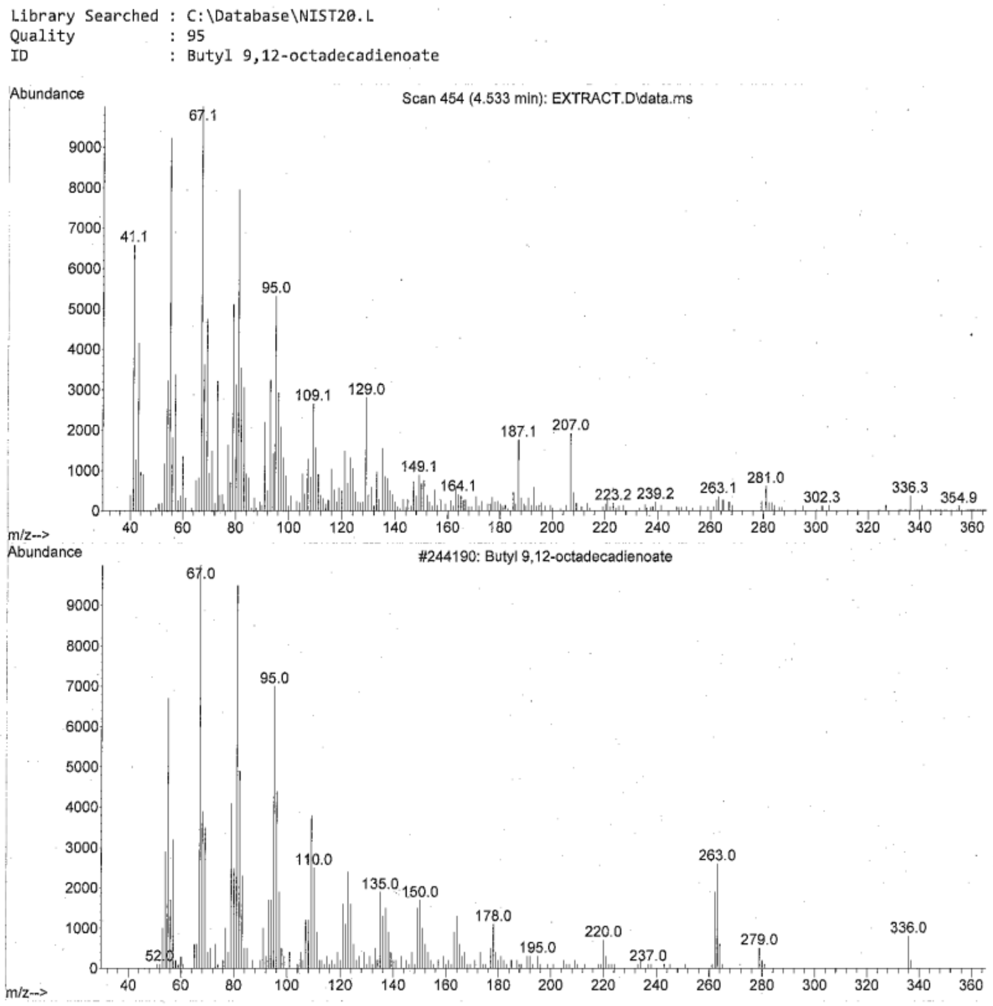

7. **Mass spectrum matched with palmitin [hexadecanoic acid, 2-hydroxy-1-(hydroxymethyl)ethyl ester].**

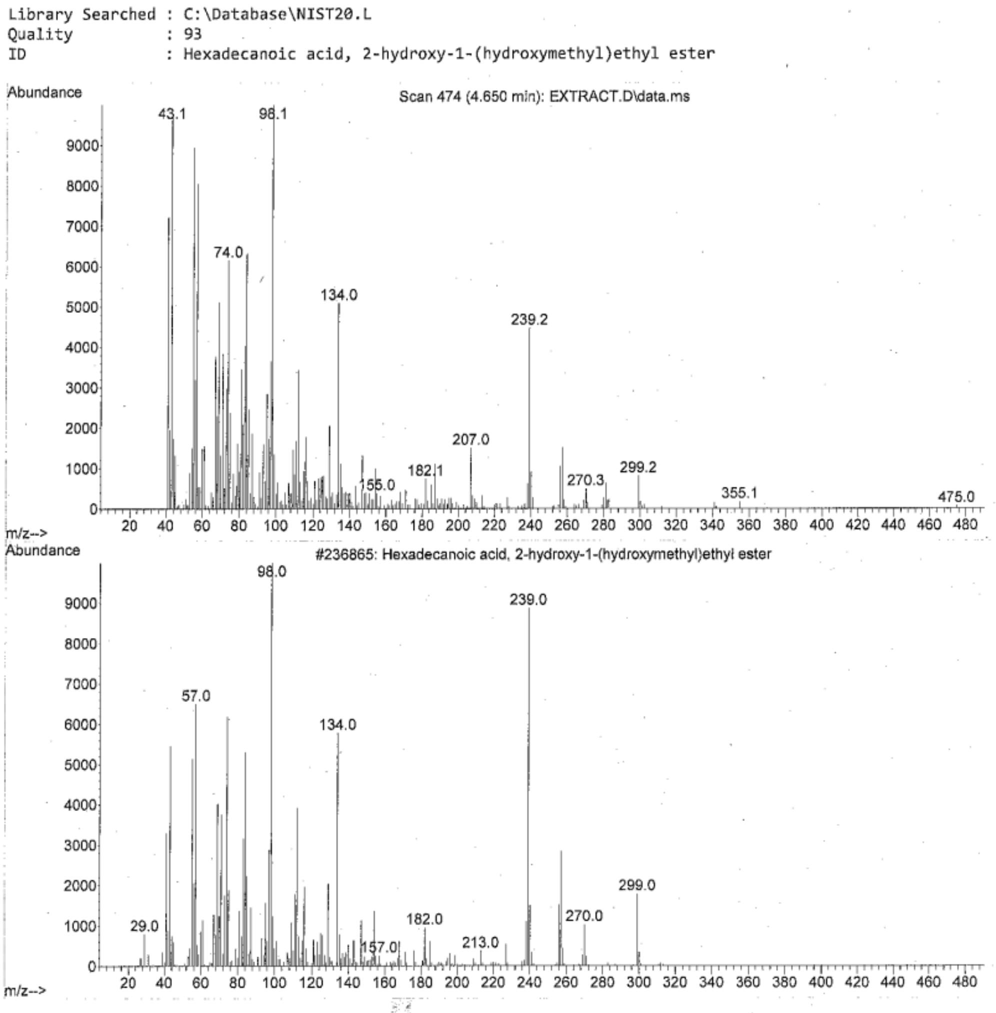

8. Mass spectrum matched with linolein [9,12-octadecadienoic acid (Z, Z)-, 2-hydroxy-1-(Hydroxymethyl)ethyl ester].

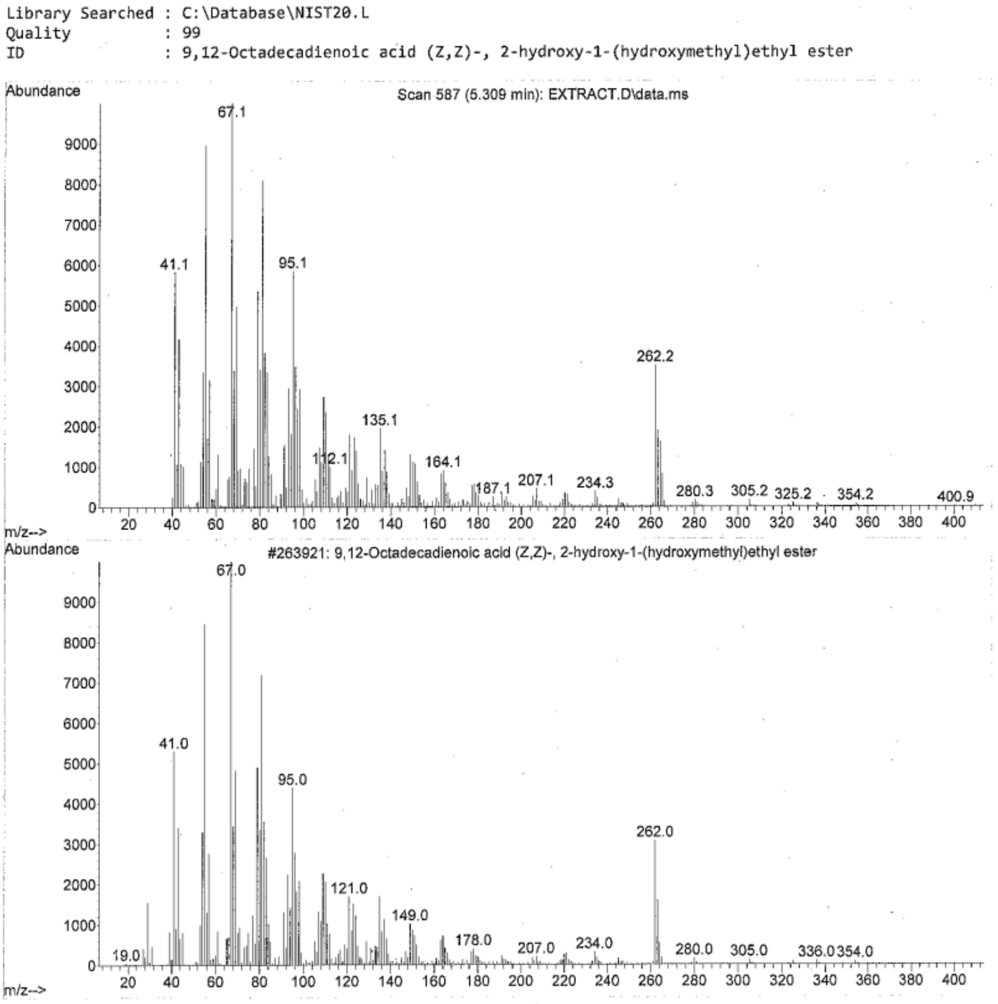

9. Mass spectrum matched with squalene.

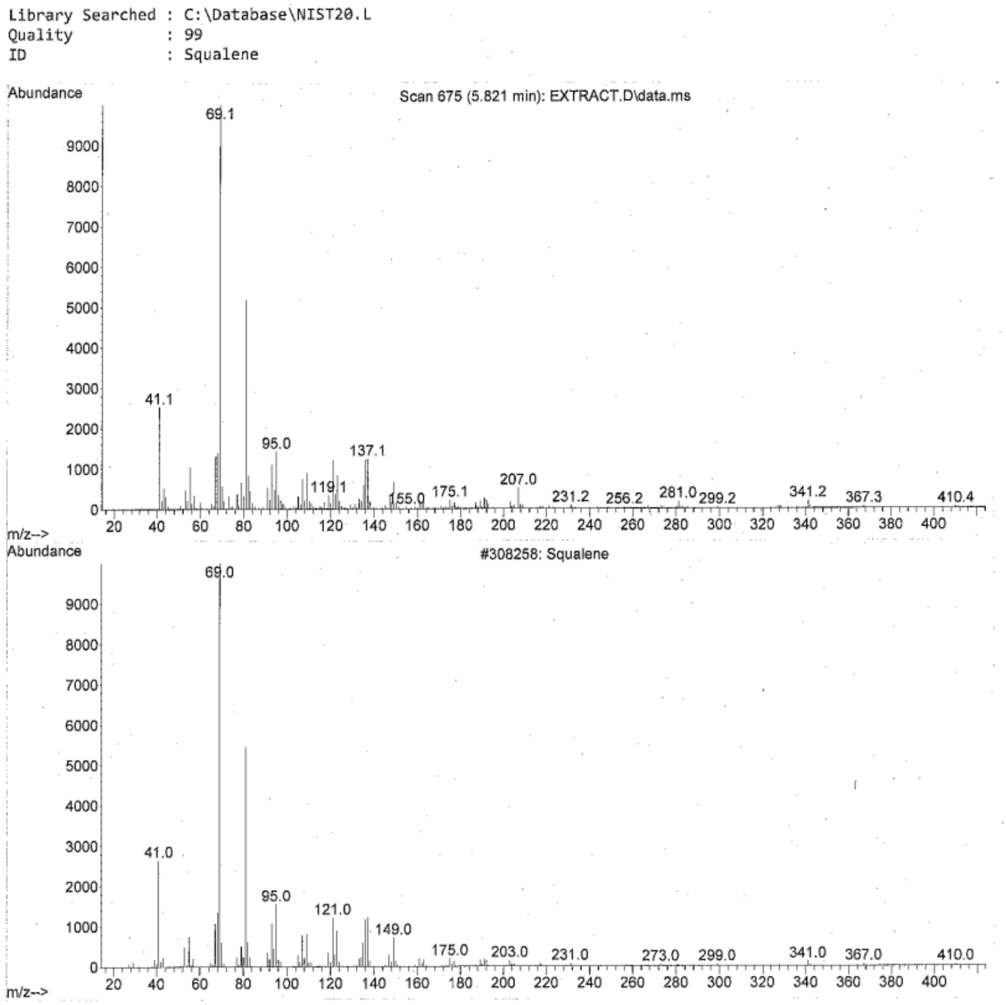

10. Mass spectrum matched with cholesterol.

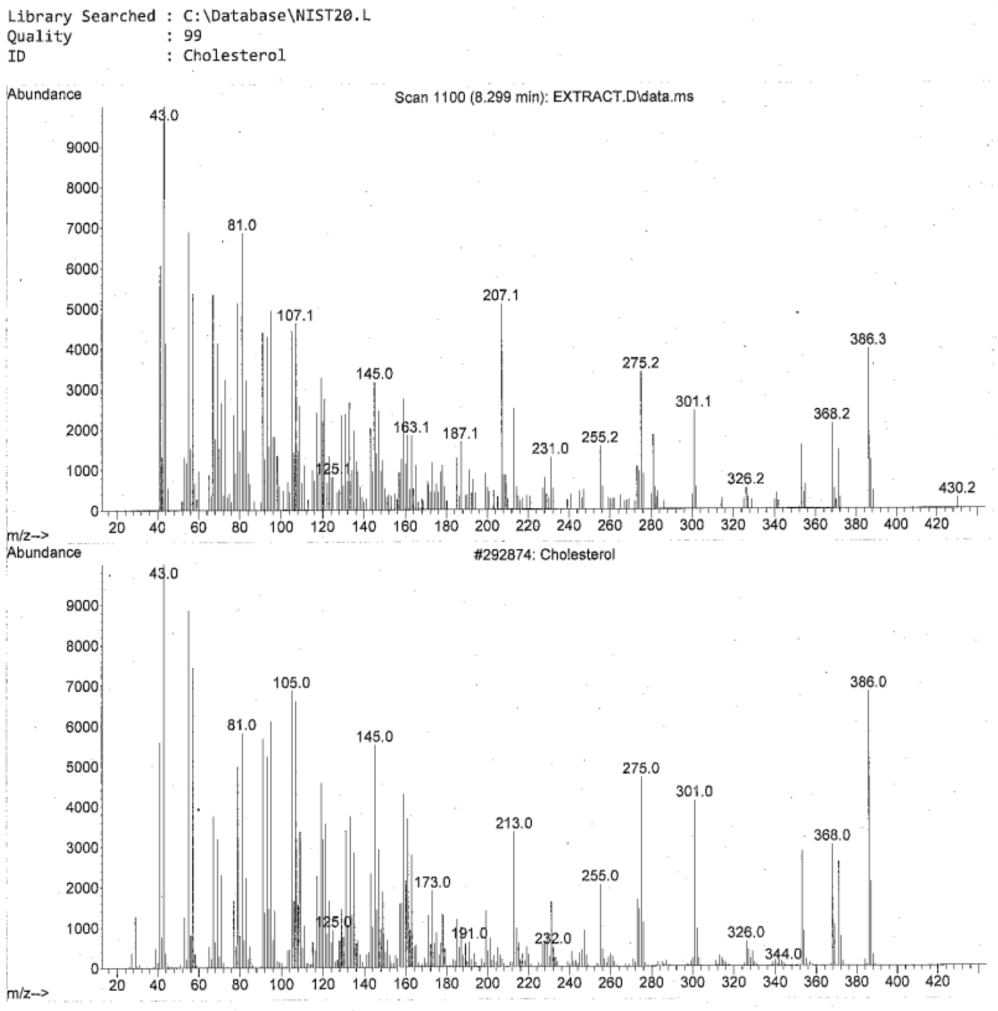

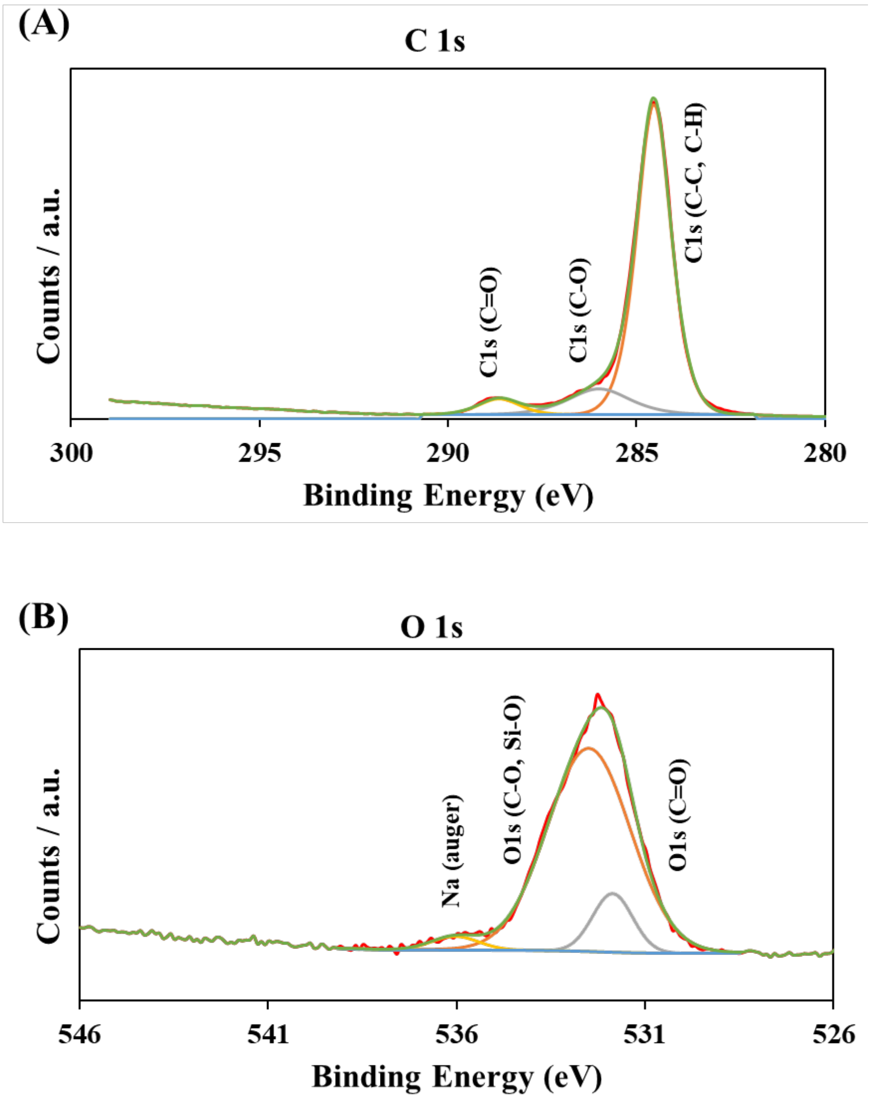

Figure S2. Deconvoluted (A) C 1s and (B) O 1s XPS spectra of CD-micelles.

.

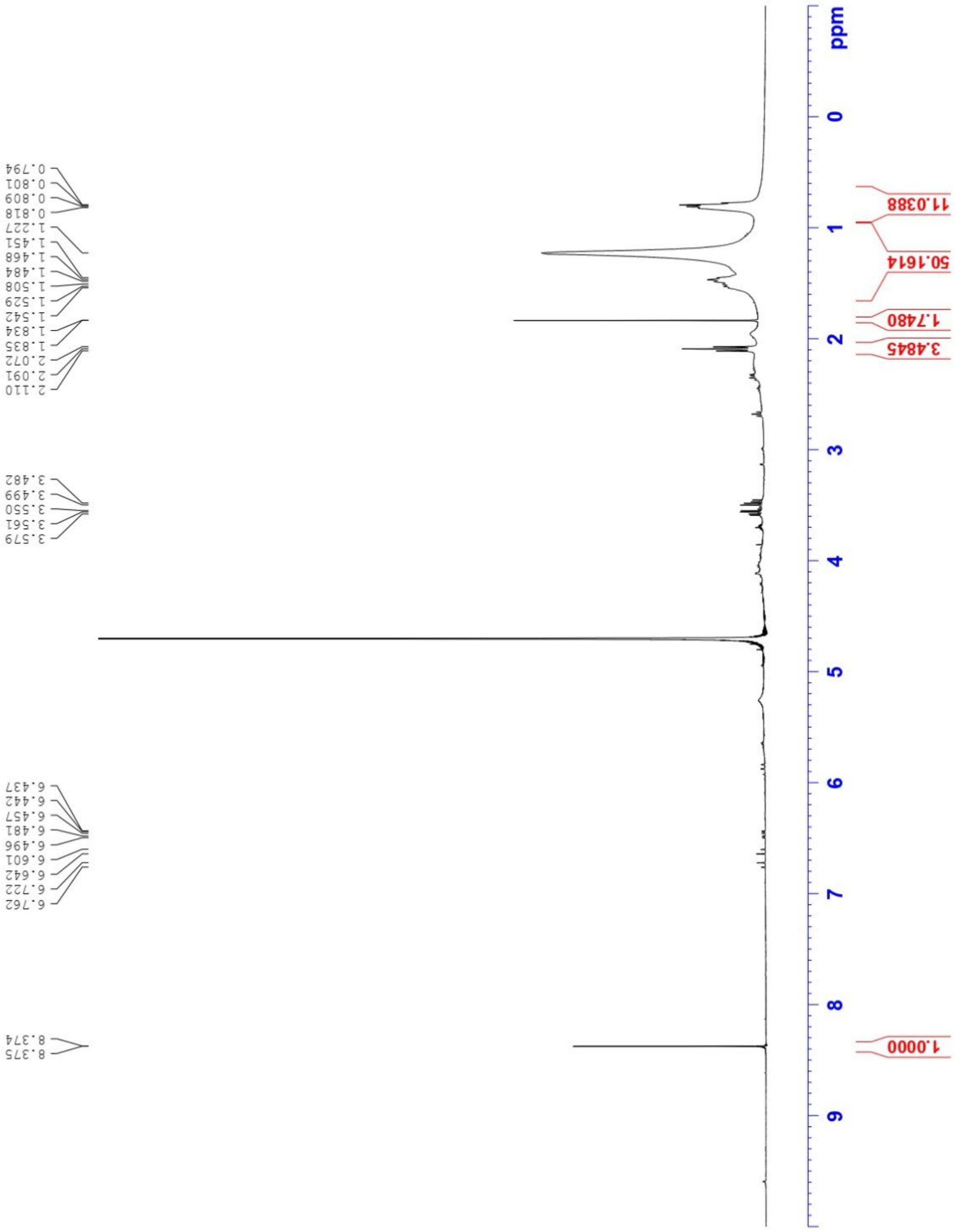

Figure S3. ^{1}H NMR spectrum of the CD-micelles

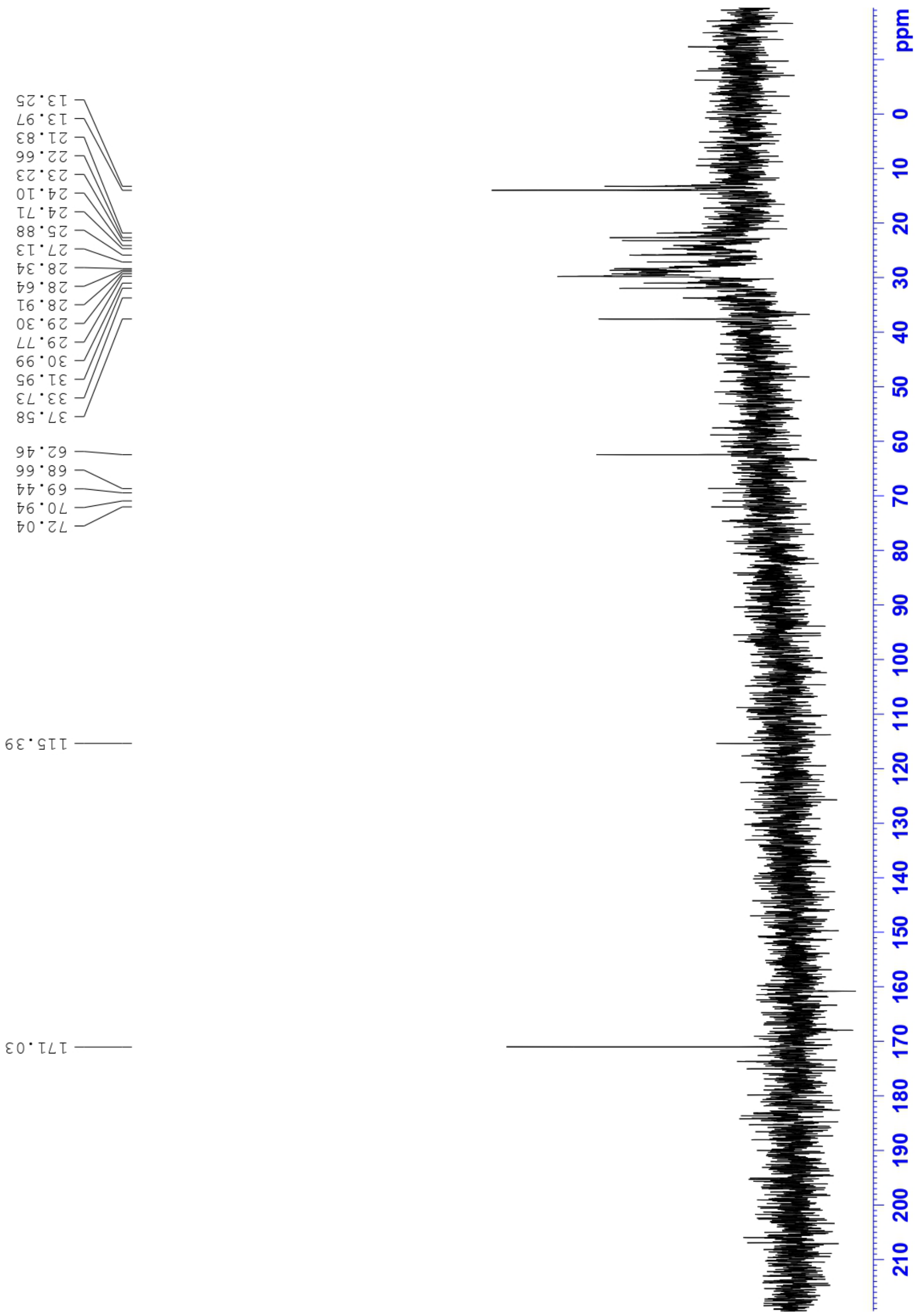

Figure S4. ^{13}C NMR spectrum of the CD-micelles

.

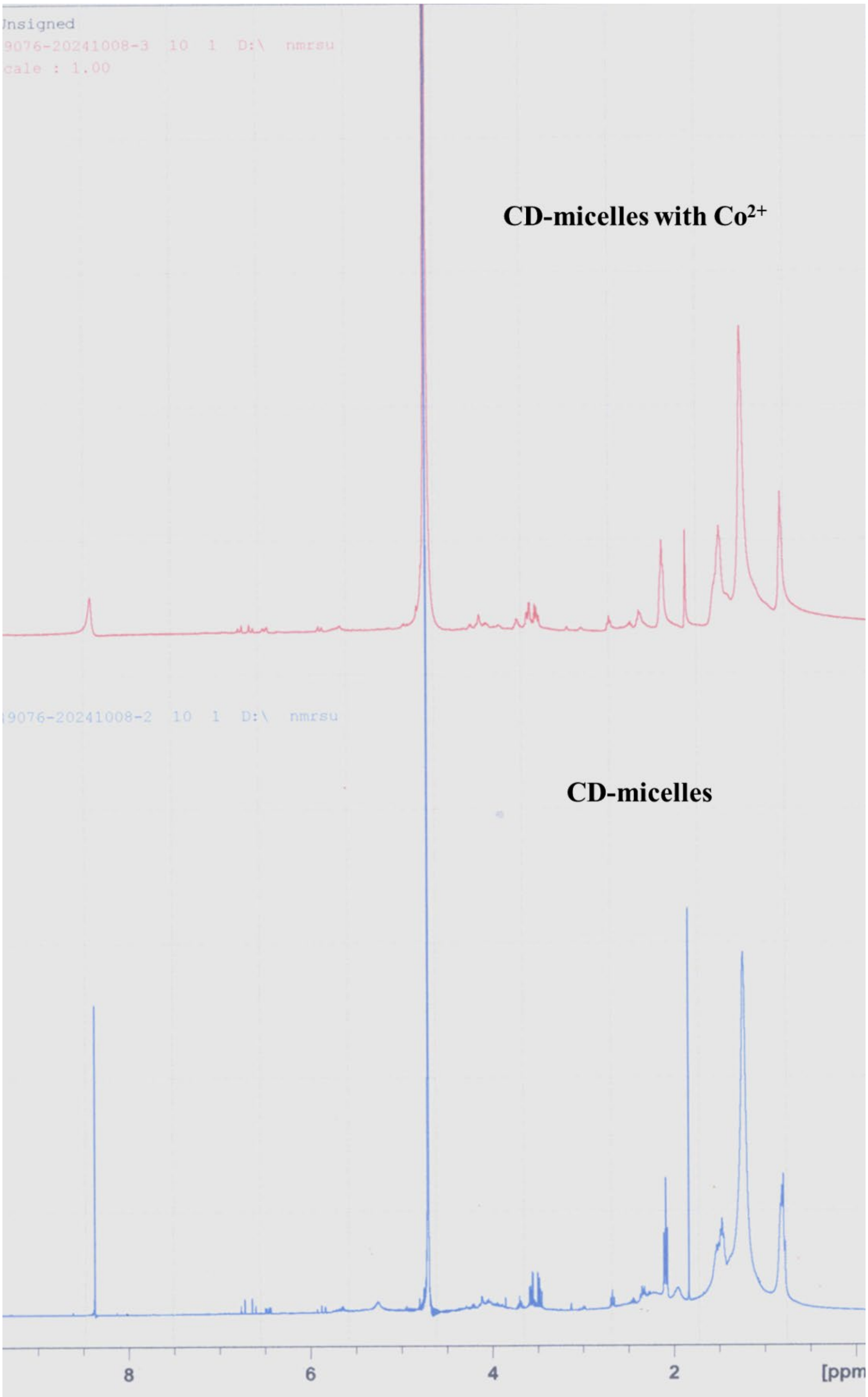

Figure S5. ^{1}H NMR spectra of the CD-micelles without (down) and with Co^{2+} (up).
